# Dioxonaphthoimidazoliums AB1 and YM155 disrupt phosphorylation of p50 in the NF-κB pathway

**DOI:** 10.18632/oncotarget.7299

**Published:** 2016-02-10

**Authors:** Si Han Sherman Ho, Azhar Ali, Tan Min Chin, Mei Lin Go

**Affiliations:** ^1^ Department of Pharmacy, Faculty of Science, National University of Singapore, Singapore; ^2^ Cancer Science Institute, Yong Loo Lin School of Medicine, Singapore

**Keywords:** non-small cell lung cancer, YM155, survivin suppressant, NF-κB signaling, p50 phosphorylation

## Abstract

The NF-κB pathway is overexpressed in non-small cell lung cancers (NSCLC) and contributes to the poor prognosis and high mortality characterizing this malignancy. Silencing the p50 and p65 NF-κB subunits in the NSCLC H1299 cell line led to profound loss in cell viability and downregulated anti-apoptotic proteins survivin and Mcl1. We also showed that a survivin suppressant, the dioxonaphthoimidazolium YM155, and its structural analog AB1 arrested the growth of H1299 cells at nanomolar concentrations. Both compounds were apoptogenic and suppressed survivin and other anti-apoptotic proteins (Mcl1, Bcl-2, Bcl-xl) in a dose- and/or time-dependent manner. YM155 and AB1 did not affect the expression of key proteins (IκBα, p65, p50) involved in NF-κB signaling. Stable IκBα levels suggest that the NF-κB/IκB complex and proteins upstream of IκBα, were not targeted. Neither did the compounds intercept the nuclear translocation of the p50 and p65 subunits. On the other hand, YM155 and AB1 suppressed the phosphorylation of the p50 subunit at Ser337 which is critical in promoting the binding of NF-κB dimers to DNA. Both compounds duly impeded the binding of NF-κB dimers to DNA and attenuated transcriptional activity of luciferase-transfected HEK293 cells controlled by NF-κB response elements. We propose that the “silencing” the NF-κB pathway effected by these compounds contributed to their potent apoptogenic effects on H1299. Notwithstanding, the mechanism(s) involved in their ability to abolish phosphorylation of p50 remains to be elucidated. Taken together, these results disclose a novel facet of functionalized dioxonaphthoimidazoliums that could account for their potent cell killing property.

## INTRODUCTION

Several important physiological processes such as cell death and inflammation are regulated by the NF-κB family of DNA binding proteins [1, 2]. Members of this family comprise p65 (RelA), p50, p52, RelB and c-Rel. They have in common a Rel homology domain (RHD) which mediates DNA binding, dimerization and interaction with IκBs – the specific inhibitory factors responsible for retaining the NF-κB homo- and heterodimers in the cytoplasm. Stimuli that activate NF-κB are transmitted by IκB kinase-dependent phosphorylation which then triggers a sequence of events starting with the degradation of IκB proteins, translocation of the liberated NF-κB dimers to the nucleus and the induction or repression of gene transcription. p50/p65 is the most abundant NF-κB dimer. The p65 subunit contains a transactivation domain and oversees transcription whereas the p50 subunit which lacks a transactivation domain, functions mainly in DNA binding [3–7]. The homodimeric p50/p50 specie competes with the p50/p65 heterodimer for binding to NF-κB sites on DNA and thus represses the activation of genes controlled by p50/p65 [8, 9].

Dysregulation of NF-κB signaling has been implicated in the pathogenesis of cancer [10, 11], and resistance to treatment modalities such as chemotherapy and radiotherapy [12–14]. The NF-κB pathway has been shown to be constitutively activated in non-small cell lung cancers (NSCLC), possibly as a downstream consequence of K-Ras mutations in these tumors [15, 16]. In a mouse lung cancer model, the NF-κB target gene Timp-1 was identified as a key stimulator of tumor growth [17]. Survivin is an anti-apoptotic protein that is consistently overexpressed in a large number of malignancies but not in normal adult tissues [18–22]. In malignant tissues, survivin is linked to the suppression of apoptosis, metastasis and resistance to therapy [23]. NF-κB activation leads to survivin overexpression in several malignancies such as multiple myeloma and adult T-cell lymphoma [24, 25]. Cross-talk between p53 and NF-κB has been reported to control survivin expression [26]. In quiescent cells, p53 binds to the survivin promoter to inhibit its transcription but under conditions of replication stress, p53 induces survivin transcription [27–29] by a mechanism involving NF-κB [30]. In animal xenografts, the transcription factor inhibitor of differentiation (Id1) increases survivin levels via a NF-κB dependent mechanism [31]. These findings point to a complex interplay between NF-κB signaling and survivin in regulating the proliferation and survivability of tumor cells.

**Figure 1:**
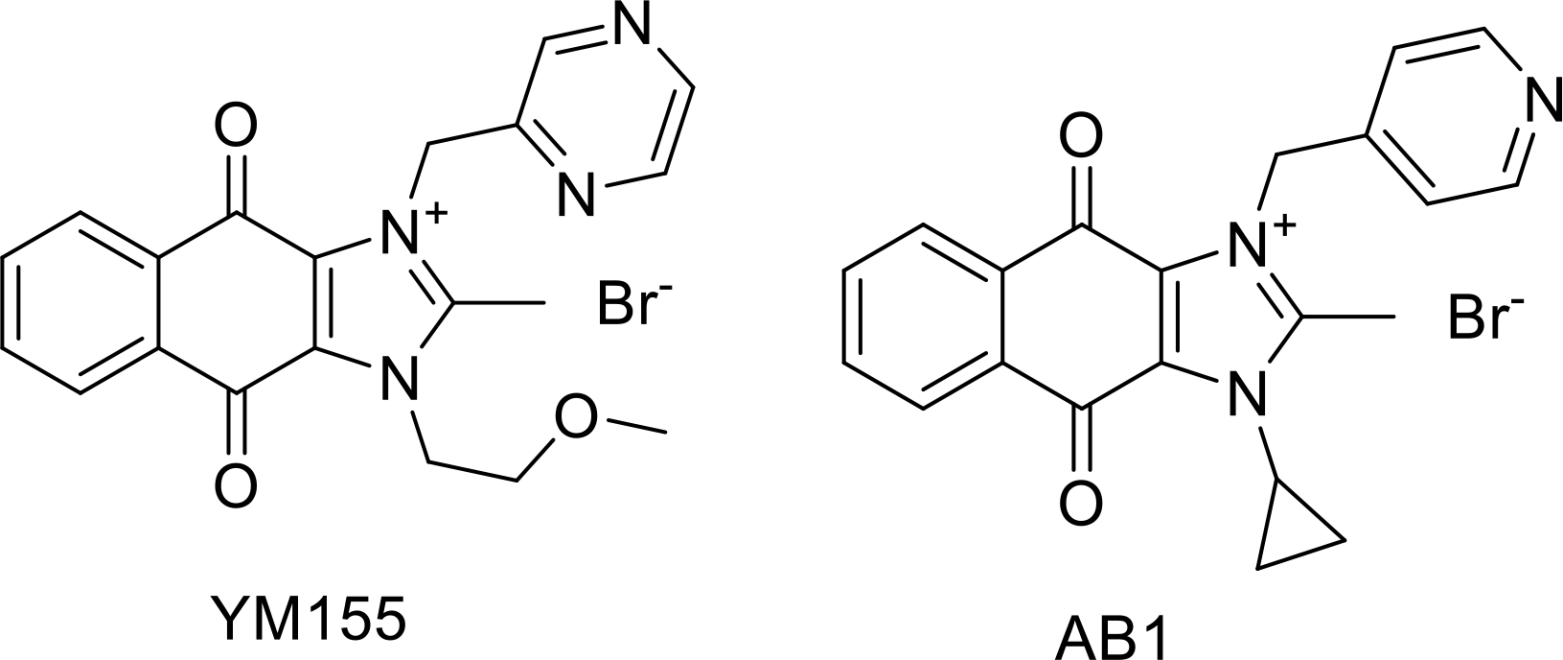
Molecular structures of the dioxonaphthoimidazoliums AB1 and YM155 investigated in this report

Although survivin has been widely cited as a viable anticancer target [32–34], few small molecule inhibitors of survivin have been reported. The most widely cited inhibitor is the dioxonaphthoimidazolium analog YM155 which acts by blocking the transcription of the survivin gene [18, 23] (Figure 1). In our investigations on the growth inhibitory activity of functionalized dioxonaphthoimidazoliums on a panel of malignant cell lines, several members were found to exhibit nanomolar growth inhibitory potencies comparable to that of YM155 [35]. The analog AB1 is one such compound (Figure 1). Strikingly, even the less active members in the library retained impressive single digit micromolar growth inhibitory activities. Clearly the dioxonaphthoimidazolium scaffold is endowed with potent cell killing properties and it would be of interest to explore the mechanistic basis undergirding its unusual activity. In view of the causality between NF-κB signaling and survivin expression, we hypothesized that disruption of the NF-κB pathway by the scaffold contributes to the suppression of survivin and cell death. To this end, investigations were carried out on an NSCLC cell line (H1299) in which the NF-κB pathway is constitutively up-regulated.

## RESULTS

### The NF-κB pathway is constitutively activated in NSCLC cells

**Figure 2:**
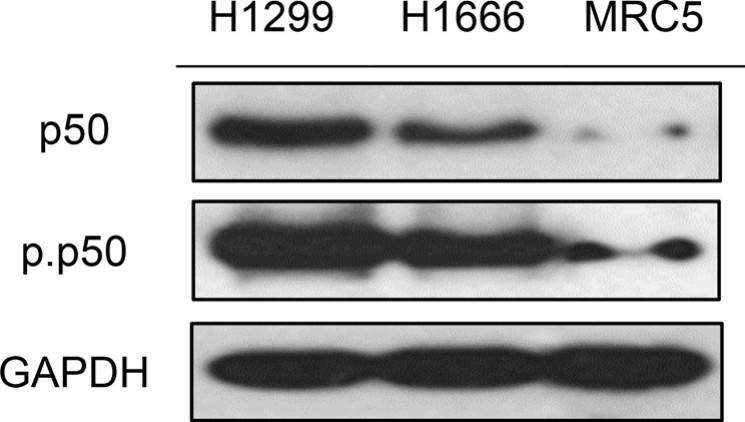
p50 and phosphorylated p50 (Ser337) are overexpressed in NSCLC cell lines H1299 and H1666 GAPDH was used as loading control.

To confirm the up-regulation of the NF-κB pathway in NSCLC, we monitored levels of the NF-κB p50 subunit and phospho-p50 (Ser337) on two NSCLC cell lines (H1299, H1666) and the normal lung cell line MRC5. Phosphorylation regulates the DNA binding affinity of p50 and serves as a marker of NF-κB activity [36, 37]. Figure 2 shows an abundance of phospho-p50 in all the NSCLC cell lines, with levels exceeding that of the non-malignant MRC5 cells. Thus, NF-kB activity is up-regulated in both NSCLC cell lines.

### The dioxonaphthoquinones YM155 and AB1 selectively inhibit the viability of NSCLC H1299 and H1666 cells

The growth inhibitory effects of AB1 and YM155 were determined on NSCLC H1299 and H1666 cells by a colorimetric tetrazolium (MTT) assay. Both cell lines are characterized by the presence of wild type EGFR but only H1299 is p53-null. As seen from Table 1, AB1 and YM155 had comparable nanomolar growth inhibitory potencies, with slightly greater potency on the p53 positive H1666 cells. Dose response curves are presented in Supplementary Information. When evaluated on non-malignant lung fibroblast IMR90 cells, both AB1 and YM155 were less potent with AB1 having a wider margin of selective activity on the NSCLC cells (17 fold) as compared to YM155 (7 to 8 fold). These results affirm the potent and selective growth inhibitory property of AB1 and YM155 on NSCLC cells. As the two compounds were nearly equipotent on both cell lines, subsequent investigations were carried out on one cell line (H1299).

**Table 1:** Antiproliferative activities (IC_50_) of AB1 and YM155 on NSCLC (H1666, H1299) and non-malignant lung fibroblast IMR90 cells

	Antiproliferative IC_50_ (nM)^a^		
	H1666	H1299	IMR90
AB1	16.8 ± 1.7	35.9 ± 3.0	287 ± 59
YM155	13.7 ± 0.9	35.5 ± 1.2	247 ± 37

a Concentration required to reduce cell viability to 50% of levels observed in untreated cells under similar experimental conditions. Determined by the MTT assay, 72 h. Mean ± SD of *n* = 3 determinations.

### YM155 and AB1 induce apoptotic cell death in H1299 cells and reduce levels of pro-survival proteins survivin, Mcl1 and Bcl-2

**Figure 3:**
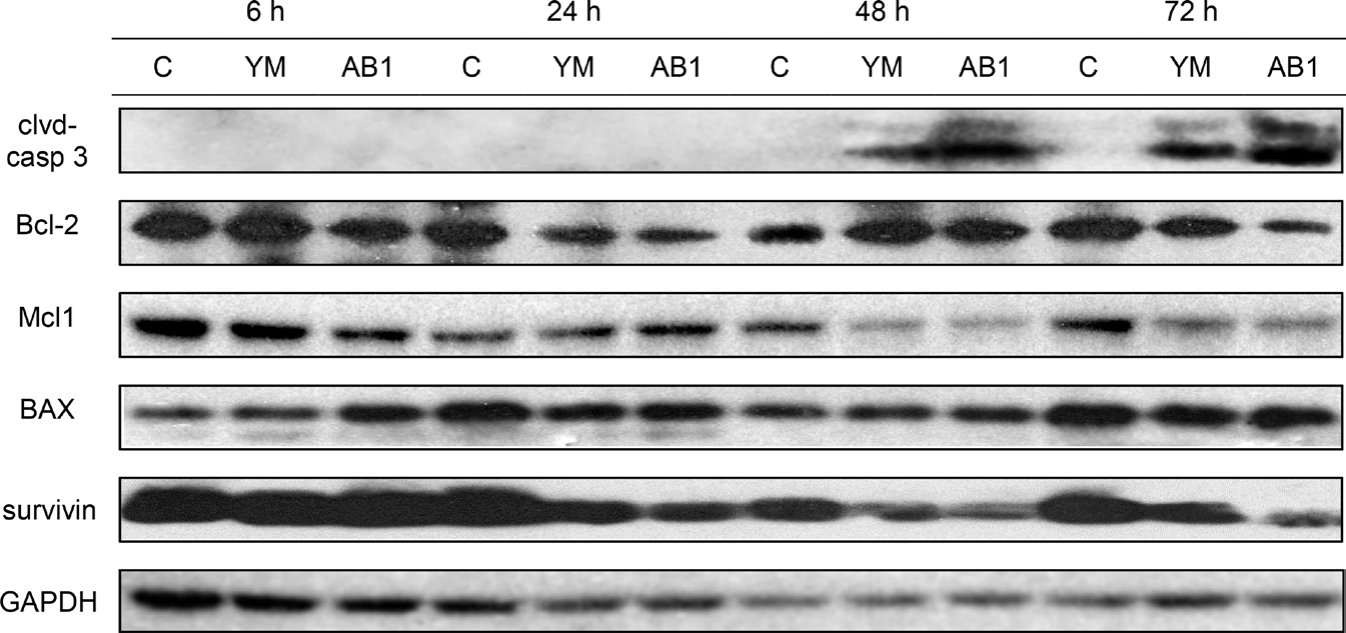
Treatment of H1299 cells with YM155 and AB1 at 38 nM which corresponds to their respective IC_50_ concentrations and corresponding time-dependent changes in cleaved caspase 3, anti-apoptotic proteins (Mcl1, survivin, Bcl-2) and pro-apoptotic BAX GAPDH was used as loading control.

**Figure 4:**
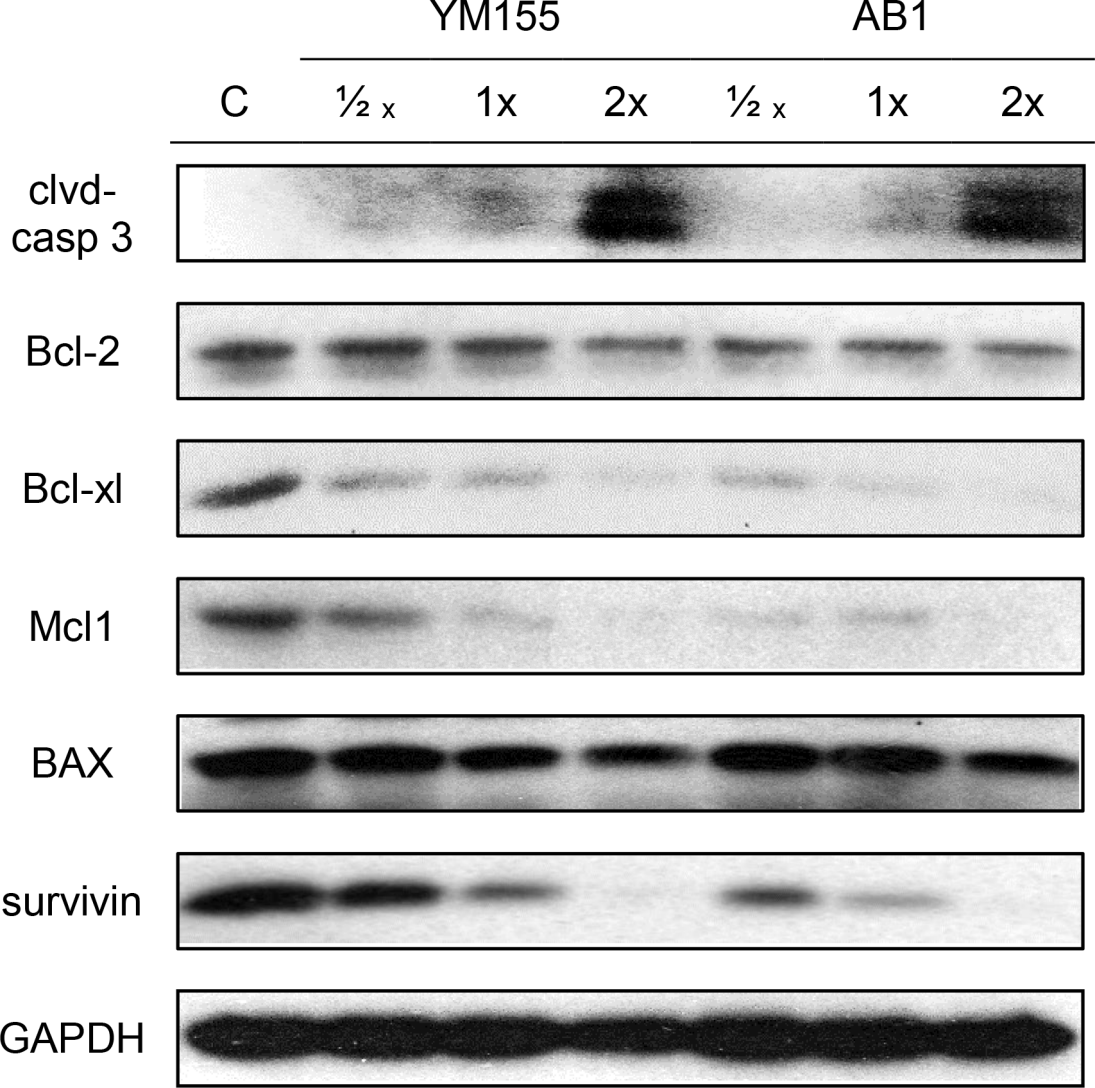
Treatment of H1299 cells with varying concentrations of YM155 and AB1 at a fixed time point of 48 h Levels of cleaved caspase 3, anti-apoptotic proteins (Mcl1, survivin, Bcl-2, Bcl-xl) and pro-apoptotic BAX were monitored. GAPDH was used as loading control.

The apoptogenic effects of YM155 have been widely reported [38–40] and it is anticipated that its structural analog AB1 should behave in like manner. To this end, we monitored the appearance of the apoptotic marker protein (cleaved caspase 3) in H1299 cells treated with YM155 and AB1 at varying time points and concentrations. Induction of apoptosis involves the conversion of procaspase 3 to its activated cleaved states which would then trigger cell-wide protein degradation [41]. Figure 3 shows time-dependent changes (6 h, 24 h, 48 h, 72 h) in cleaved caspase 3 in cells treated with AB1 and YM155 at their IC_50_ concentrations. The appearance of cleaved caspase 3 at the 48 h time point signals the onset of apoptosis in these cells. When treated with varying concentrations (0.5× IC_50_, 1× IC_50_, 2× IC_50_) of AB1/YM155 at a fixed time point of 48 h, dose-dependent increases of cleaved caspase were observed (Figure 4).

The effects of YM155 and AB1 on selected anti- and pro-apoptotic proteins were also monitored for time and dose dependencies. Of the pro-apoptotic proteins, survivin and Mcl1- but not Bcl-2 - were reduced by both compounds (investigated at IC_50_) over time (Figure 4). There was an early reduction in survivin (24 h) while that of Mcl1 was observed later (48 h). Pro-apoptotic BAX was not affected by either compound even after 72 h. The experiments were then repeated at a fixed time point (48 h) at various concentrations of YM155 and AB1 (Figure 4). BAX levels were once again not significantly perturbed by either compound, even at 2 × IC_50_. However, dose dependent decreases were observed for the pro-apoptotic proteins survivin, Mcl1, Bcl-2 and Bcl-xl. The reduction in Bcl-2 which was not observed at its IC_50_ at 48 h (Figure 3), was now apparent at the higher concentration (2 × IC_50_). Interestingly, AB1- induced losses of Mcl1 and Bcl-xl were observed at a lower concentration (0.5 × IC_50_) as compared to YM155 (1 × IC_50_).

**Figure 5:**
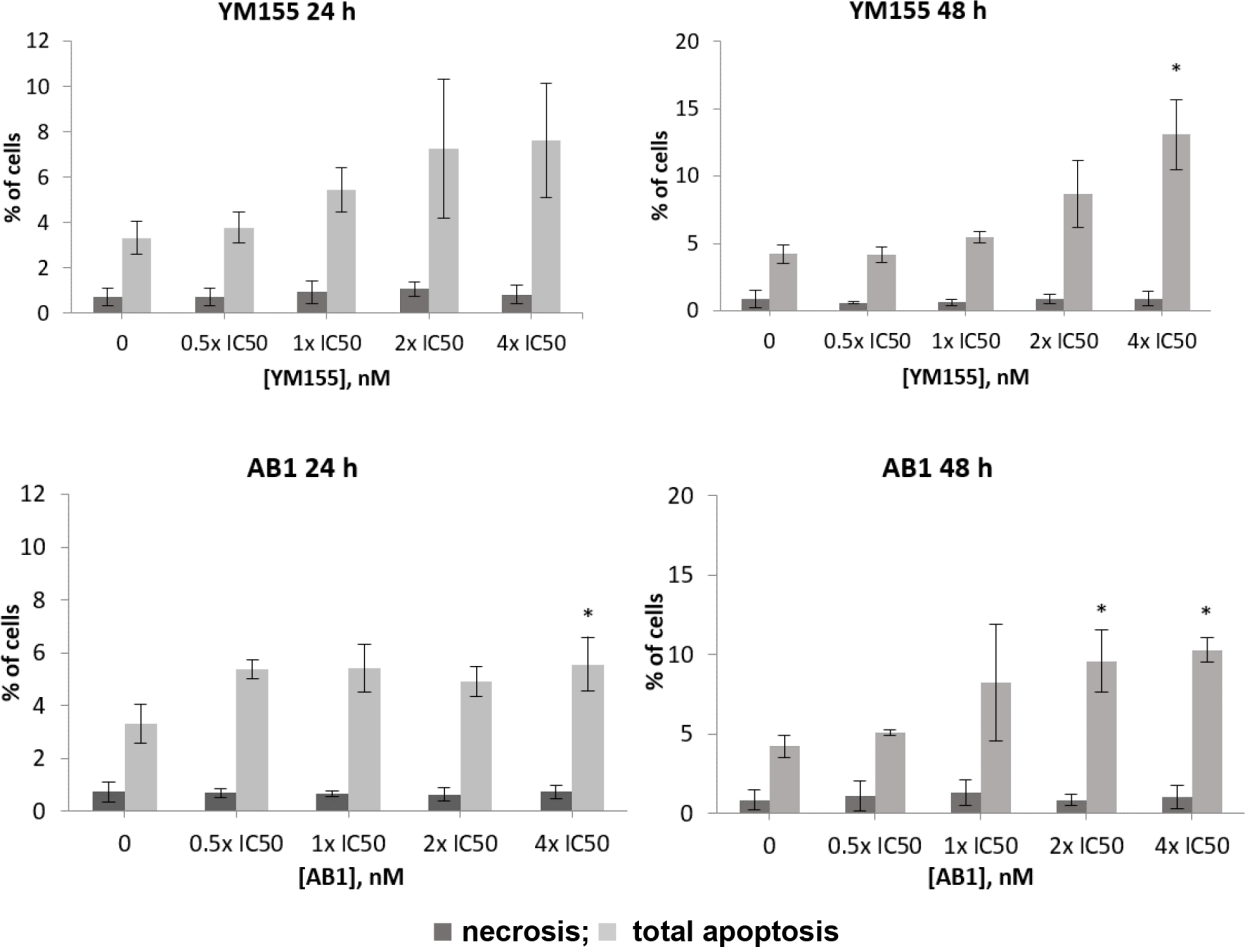
Proportion of apoptotic and necrotic H1299 cells as determined by FACS analysis after double staining with Annexin V-FITC and PI Cells were treated with different concentrations (0.5 ×, 1 ×, 2 × and 4 × growth inhibitory IC_50_) of test compound at 24 and 48 h time points. Error bars represent the standard deviations of three separate experiments. Significance difference from control is indicated by an asterisk (*p* < 0.05).

Cell death may occur by various modes, of which apoptosis is the most common. It may also occur by necrosis which unlike apoptosis, is un-programmed and induced by cellular damage. YM155 has been cited to cause DNA damage [26] which could be a trigger for necrotic cell death. Although we have shown that YM155 and AB1 were apoptogenic, it was of interest to determine if necrosis is also involved in the cell death process. To this end, H1299 cells were treated sequentially with varying concentrations of YM155 or AB1 for 24 h or 48 h, followed by double staining with Annexin V-FITC (fluorescein isothiocyanate) conjugate and propidium iodide (PI), and subsequently, analysis by FACS. Briefly, the method is based on the translocation of phosphatidylserine from the inner to outer surface of cell membranes upon induction of apoptosis. Once positioned in the outer membrane, the phosphatidylserine residues bind to Annexin V and are detected by the fluorescence of FITC. On the other hand, PI (a DNA intercalator) is excluded from viable cells and only permeates cells in late apoptosis or necrosis. Thus, apoptotic cells are positively stained by Annexin V but not PI (early apoptosis) or positively stained by both Annexin V and PI (late apoptosis). Necrotic cells are only stained by PI [42]. To assess the relative contributions of apoptosis and necrosis to the cell death phenomenon, we quantified the proportion of cells that were stained by PI (proxy for necrosis) and those stained by Annexin V (indicative of cells in early and late apoptosis). As seen from Figure 5, neither YM155 nor AB-1 significantly altered the proportion of necrotic cells over time or at different concentrations. In contrast, the proportion of apoptotic cells increased with time although significant increases were only observed at 4 × IC_50_ of either compound. Notwithstanding, the proportion of apoptotic cells exceeded that of necrotic cells at each treatment condition, indicating that apoptosis is the main driver of cell death by YM155 and AB1. That concentrations exceeding the IC_50_ were required to induce apoptosis is attributed to the assay methodology which involved shorter incubation times (48 h versus 72 h in the growth inhibitory assay), and higher cell densities.

Taken together, we have shown a close alignment in the apoptogenic effects of YM155 and AB1 on H1299 cells. Both compounds decreased expression of the pro-apoptotic proteins (survivin, Mcl1, Bcl-2, Bcl-xl) but did not induce a concurrent increase in the anti-apoptotic BAX protein. Noting that Mcl1, Bcl2 and Bcl-xl suppress apoptosis at the mitochondrial level which precedes caspase activation, apoptosis induced by YM155 and AB1 is likely to be independent of caspase activity. On a related note, the suppression of Mcl1 by YM155 has been investigated in some detail and found to occur at the transcriptional level, independently of survivin expression and caspase activity [43].

### Canonical NF-κB members p50 and p65 are essential for H1299 viability

Having shown that the NF-κB pathway is constitutively activated in H1299 cells (Figure 2), we proceeded to assess the importance of the p50 and p65 subunits to cell viability. To this end, H1299 cells were transfected with siRNAs targeting the p50 or p65 gene. The p50 siRNA was evaluated at 10 nM or 50 nM and cell viability was assessed at 48 h and 72 h time-points. Silencing of p65 was explored under similar conditions.

**Figure 6:**
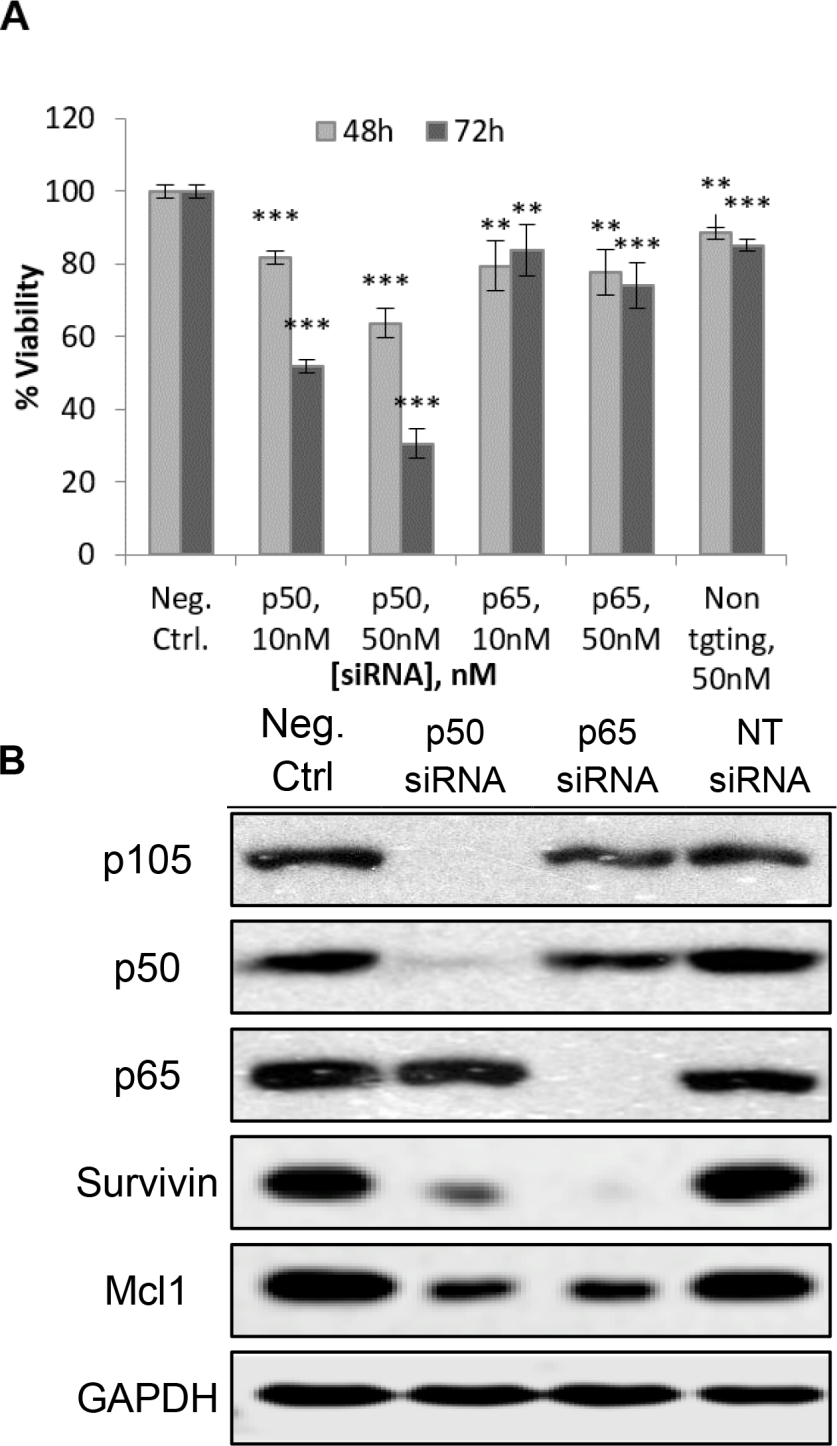
(**A**) Silencing of p50 and p65 led to a loss of cell viability of H1299, with greater losses observed on the silencing of p50 than p65. *p < 0.05; **p < 0.01; ***p < 0.001. (**B**) Western blotting showing successful silencing of p50 and p65 in H1299 72 h post-transfection. p105 is the precursor form of p50 and is cleaved to give p50. Survivin and Mcl1 were also suppressed with silencing of p50 and p65. All siRNA were used at 50 nM. NT siRNA = Non-targeting siRNA.

Cell viability was significantly decreased by the silencing of the p50 gene in H1299 cells (Figure 6A). The losses increased with time (72 h > 48 h) and dose of siRNA (50 nM > 10 nM). In contrast, smaller decreases in cell viability were observed when the p65 gene was silenced and there was limited evidence of dose or time dependency. The effects of silencing p50 and p65 on the expression of anti-apoptotic survivin and Mcl1 were also investigated (Figure 6B), It was found that silencing p50 or p65 caused a profound reduction in the expression of both proteins, affirming the role of the NF-κB pathway in the regulation of survivin and Mcl1 in H1299 cells.

### AB1 and YM155 inhibit phosphorylation of p50 but not p65 in H1299 cells

**Figure 7:**
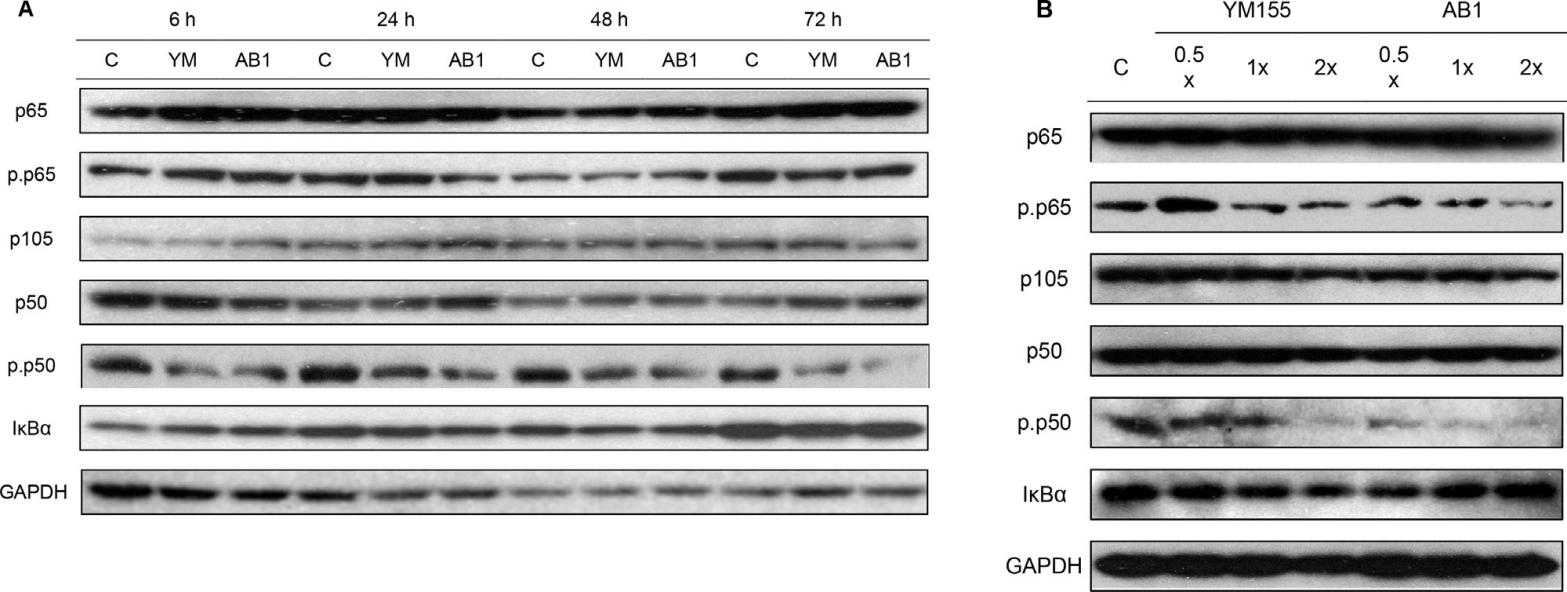
(**A**) Treatment of H1299 with YM155 and AB1 at 38 nM which corresponds to their respective IC_50_ concentrations and corresponding time-dependent changes in total and phosphorylated NF-κB subunits and IκBα. (**B**) 48 h treatment of H1299 with YM155 and AB1 at 0.5 ×, 1 × and 2 × IC_50_ and corresponding dose-dependent changes in total and phosphorylated NF-κB subunits and IκBα. GAPDH was used as loading control in both sets of blots.

In view of the importance of the p50 subunit in regulating the viability of H1299 cells (Section 2.1), it was of interest to determine the effects of YM155 and AB1 on the expression levels of p50, its precursor protein p105, p65 and the inhibitory protein IκBα. These proteins were probed by western blotting in cells treated with YM155 and AB1 at their IC_50_ over 6 h to 72 h. (Figure 7A). No change in their levels were noted. Levels also remained unchanged when cells were treated with increasing amounts (½ × IC_50_ to 2 × IC_50_) of YM155 and AB1 for 48 h (Figure 7B).

Both p50 and p65 are functionally activated by phosphorylation. Phosphorylation of p65 at Ser536 controls its transcriptional activity while phosphorylation of p50 at Ser337 promotes DNA binding [36, 37, 44]. Hence, levels of phospho-p65 (Ser536) and phospho-p50 (Ser337) were monitored in treated cells over time (Figure 7A) and at varying concentrations of AB1/YM155 (Figure 7B). Neither compound affected the levels of phospho-p65 in a time dependent manner although phospho-p65 levels were diminished at higher concentrations (2 × IC_50_) of both compounds. In stark contrast, phospho-p50 levels were markedly reduced by both AB1 and YM155. The decreases were observed early (6 h) and were more pronounced with time. Dose dependent suppression of phospho-p50 was also observed, with AB1 completely suppressing phospho-p50 at its IC_50_.

From these results, we deduced that AB1 and YM155 do not disrupt the expression of the key proteins – IκBα, p65, p50 and its precursor p105 – involved in NF-κB signaling. That IκBα levels remained unchanged was notable as it would suggest that the NF-κB/IκB complex was not targeted. Rather, the compounds selectively intercepted the phosphorylation of p50 which is required for the binding of the NF-κB dimers (p50/p50, p50/p65) to DNA. Conversely, phosphorylation of p65 was not impeded by YM155 and AB1.

### AB1 and YM155 reduced p50 binding to its consensus elements without affecting translocation into the nucleus

Having shown that AB1 and YM155 suppressed phosphorylation of p50, we asked if this would have observable effects on the binding of p50/p50 and p50/p65 dimers to their DNA consensus elements since binding is dependent on p50 phosphorylation. To investigate this possibility, we used a commercially available enzyme-linked immunosorbent assay-based kit. Briefly, nuclear extracts were prepared and added to a 96 well plate to which oligonucleotides corresponding to the κB consensus site were immobilized. Once bound to the κB site, the NF-κB subunit was detected by its primary antibody (p65 or p50), followed by a HRP-conjugated secondary antibody to provide a chemiluminescent signal which measured the extent of binding.

**Figure 8:**
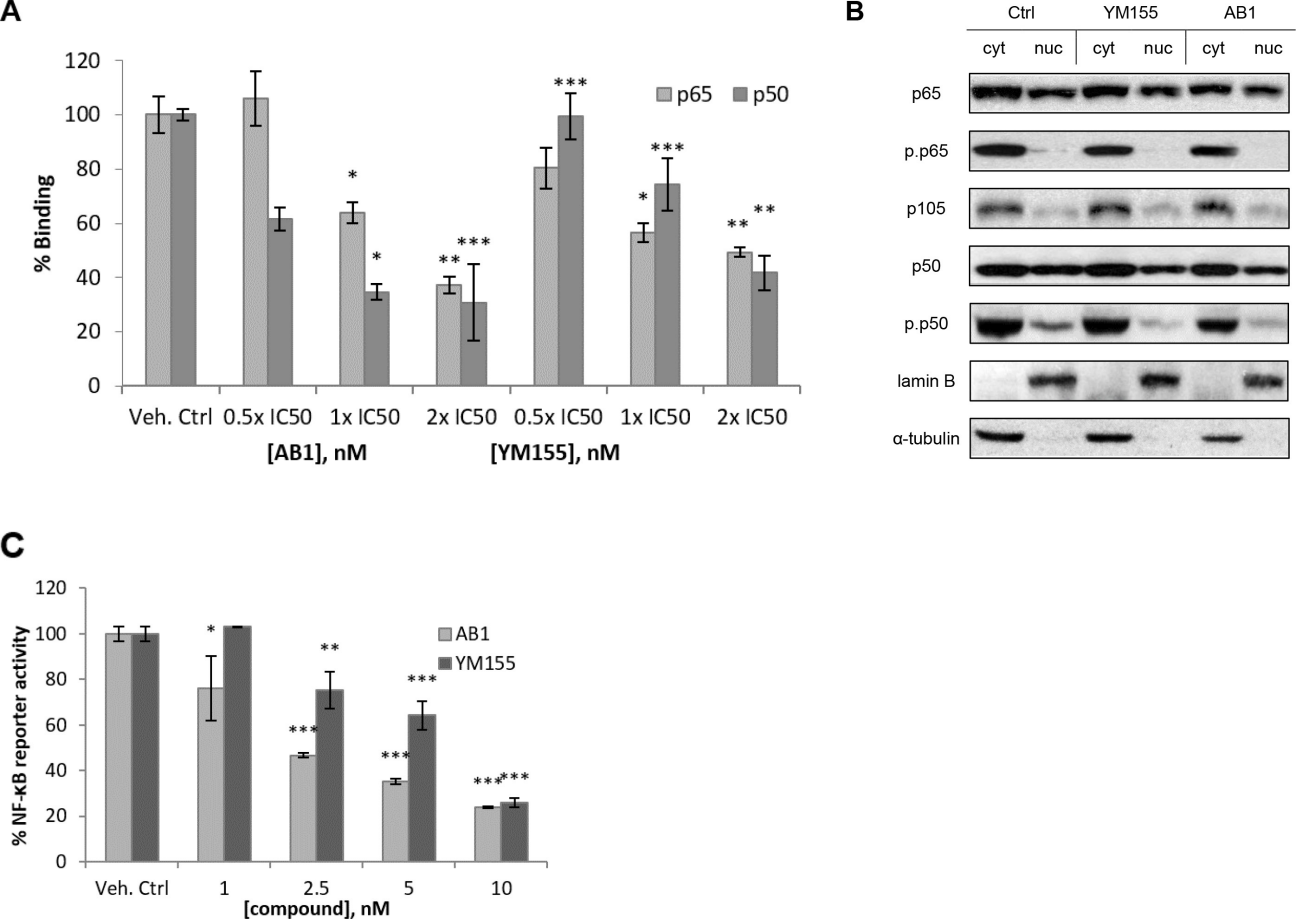
(**A**) Treatment of H1299 with AB1 and YM155 resulted in a loss of binding of p65 and p50 to the consensus NF-κB response element DNA sequence. *p < 0.05; **p < 0.01; ***p < 0.001. (**B**) Cytoplasmic and nuclear fractions of H1299 and 786-0 showed reduction in phosphorylated p50 after 48 h treatment with YM155 or AB1 at their respective IC_50_. (**C**) NF-κB promoter activity was significantly decreased in the presence of YM155 or AB1. *p < 0.05; **p < 0.01; ***p < 0.001.

YM155 and AB1 induced dose-dependent reductions in the binding of p50 and p65 to their consensus elements (Figure 8A). The decreases, in particular binding to p50, were more pronounced for AB1 than YM155.

Next, we considered the possibility that a reduction in DNA binding could be due to impeded translocation of the NF-κB dimers from cytosol to nucleus. To investigate this possibility, H1299 cells were treated with AB1 and YM155 (IC_50_, 48 h), the cytosolic and nuclear fractions were isolated and probed by western blotting for total and phosphorylated p65 and p50. As shown in Figure 8B, p50 and p65 were found in the cytosolic and nuclear compartments of untreated cells and treatment with YM155 or AB1 did not perturb p50 or p65 levels in either compartment. Thus, it is unlikely that these compounds intercepted nuclear translocation of NF-κB dimers (p50/p65, p50/p50). Turning to the phosphorylated species, we found phospho-p65 to be almost entirely localized in the cytosol of untreated cells and levels were largely unchanged in the presence of AB1 or YM155 (Figure 8B). In contrast, phospho-p50 was found in both cytosolic and nuclear extracts of untreated H1299 cells, with higher levels in the cytosol. Exposure to AB1 or YM155 caused significant reductions in nuclear phospho-p50 whereas cytosolic phospho-p50 levels were reduced to a lesser extent. Taken together, these results support the view that AB1 and YM155 did not block the translocation of p65 and p50 subunits to the nuclear compartment but exclusively suppressed the phosphorylation of p50 which then led to the decreases in DNA binding observed in Figure 8A.

Thus, YM155 and AB1 (i) did not intercept the cytosol to nuclear translocation of NF-κB dimers; (ii) reduced the phosphorylation p50 (more apparent in the nucleus) and (iii) reduced binding of nuclear p50 to its κB consensus elements.

### YM155 and AB1 reduce expression of NF-κB controlled genes in transfected HEK293 cells

Reduced p50 binding to the κB consensus elements would result in diminished transcription of NF-kB controlled genes. This was investigated in a luciferase reporter assay in which HEK293 cells containing a stably transfected luciferase gene under the control of a series of NF-κB response elements were incubated with increasing amounts (1 – 10 nM) of YM155 and AB1 for 48 h. TNFα was then added to stimulate the pathway. If transcription was interrupted, a diminished luminescent signal would be detected. Figure 8C shows that both compounds reduced NF-κB reporter activity. AB1 was more potent than YM155 in this regard as it elicited a 50% reduction at 2.5 nM as compared to 25% reduction at the same concentration of YM155. Thus, there is a strong likelihood that YM155 and AB1 attenuated transcriptional activity and reduced the expression of NF-κB controlled genes. Experiments on transfected NSCLC cell lines will provide the necessary confirmation.

## DISCUSSION

The anticancer activity of YM155 has been reported in the literature for almost a decade since its discovery in a high-throughput survivin gene promotor assay [45]. However, its role as a selective survivin suppressor has been increasingly questioned [26], with a widely cited claim that YM155 acts primarily as a DNA damaging agent [46]. Others have shown that YM155 reduced the transcription and expression of anti-apoptotic Mcl1 [43, 47, 48], suppressed EGFR signaling [49, 50], and reduced the levels of other oncogenic proteins including PI3K, ERK, STAT3 and securin [49, 51]. These findings suggest that YM155 has other bona fide targets apart from survivin.

Two main findings have emerged from the present investigations. First, many of the properties reported for YM155 are not exclusive to the molecule per se but are common to the dioxonaphthoimidazolium scaffold. This is shown by the close alignment in the activity profiles of YM155 and AB1 in our investigations. Second, we have uncovered a hitherto unrecognized property of dioxonaphthoimidazoliums on p50 phosphorylation. Both AB1 and YM155 induced dose- and time-dependent losses in phospho-p50 in H1299 cells. Intriguingly, p50 and p65 levels in the cytosol and nuclei of treated cells were maintained at the same levels found in untreated cells, thus lending support to the notion that translocation of the NF-κB dimers from cytosol to nucleus was not intercepted by the test compounds. Furthermore, IκBα levels were unchanged, implying that the NF-κB/IκB complex and by extension, proteins that lie upstream of IκBα in the signaling pathway, were not affected by AB1 and YM155.

AB1 and YM155 inhibited the binding of both p50 and p65 to the κB consensus sequences and attenuated transcriptional activity in transfected HEK293 cells which would imply a concurrent loss in the modulatory roles of the NF-κB subunits. Notably, genes that were activated by p50/p65 would be repressed while those that were repressed by p50/p50 would now be activated. AB1 and YM155 were in effect “silencing” the NF-κB pathway and the consequence, as seen from the silencing experiments of the p50 gene, was a significant loss in cell viability.

Notwithstanding, the question remains as to how YM155 and AB1 suppressed the p50 phosphorylation. Various kinases have been proposed to effect phosphorylation of p50. It has been posited that a protein kinase A subunit was responsible for phosphorylating Ser337 of p50 [36] while others have proposed phosphorylation at alternative serine residues in p50 by a DNA-dependent protein kinase, which oversees both DNA binding and transcriptional activity [52]. It is conceivable that AB1 and YM155 suppressed the activities of these or other putative enzymes involved in p50 phosphorylation. However, AB1 was not found to inhibit a panel of 97 kinases representing members from serine, threonine and tyrosine families (ScanEDGE^SM^ Kinase Assay by DiscoveRx). Among the kinases screened were IKK and protein kinase A (Supplementary Information, Table 1). Thus, the means by which YM155 and AB1 suppressed p50 phosphorylation remains an open question.

The NF-κB pathway is closely linked to several proteins affected by YM155 and AB1. Notably, the anti-apoptotic proteins Mcl1, Bcl-xl and survivin which were downregulated by both compounds are controlled in part by the NF-κB pathway [53–55]. Thus, the inhibition of p50 phosphorylation by the dioxonaphthoimidazoliums may be an initial event leading to the suppression of downstream oncogenic pathways and culminating in apoptotic cell death.

## MATERIALS AND METHODS

### General biology

MTT [3- (4, 5-dimethylthiazol-2-yl)-2, 5-dipheny ltetrazolium bromide] was purchased from Alfa Aesar, Inc. (Lancashire, United Kingdom), reconstituted in PBS to 2 mg/mL and diluted with appropriate cell culture media before use. Lipofectamine 3000^®^, TNFα and ON-TARGETplus^®^ non-targeting siRNAs were obtained from Life Technologies Inc (Carlsbad, CA, USA), Peprotech, (Rocky Hill, NJ, USA) and GE Dharmacon^®^ (Little Chalfont, Buckinghamshire, UK) respectively. Annexin V-FITC apoptosis detection kit and Cellytic M^®^ buffer were from Sigma-Aldrich (St. Louis, MO, USA). Pierce transcription factor assay kits for NF-κB p65/p50 and NE-PER^®^ nuclear and cytoplasmic extraction reagents were from Thermo Scientific (Rockford, IL, USA). All antibodies were from Cell Signaling Technology Inc. (Danvers, MA, USA) except for anti-GADPH and anti-p50 (Ser337) antibody which were from Santa Cruz Biotechnology Inc. (Santa Cruz, CA, USA). Product codes of primary antibodies are as follows: cleaved caspase-3 (#9664); Bcl-2 (#2872); Mcl1 (#4572); Bcl-xl (#BAX (#2772); survivin (#2803); p65 (#8242); p65 Ser536 (#3031); p105/50 (#3035); p50 Ser337 (sc-33022); IκBα (#4812); GAPDH (sc-48166). p50 or p65 siRNA were also from Santa Cruz. ONE-Glo™ Luciferase assay reagent was from Promega (Madison, WI, USA).

### Cell culture

Human NSCLC cell line H1299, H1666 and normal lung fibroblasts IMR90, MRC5 cells were from American Type Culture Collection (ATCC, Rockville, MD, USA). GloResponse^™^ NF-κB–RE-*luc2P* HEK293 cell line was from Promega (Madison, WI, USA). IMR90 cells were cultured in DMEM supplemented with 10% fetal bovine serum (FBS). MRC5 cells were cultured in MEM supplemented with 10% FBS. H1299 and H1650 cells were cultured in RPMI 1640 supplemented with 10% FBS and GloResponse^™^ HEK293 cells were grown in DMEM supplemented with 50 μg/mL hygromycin B from Sigma-Aldrich and 10% FBS. Cell lines were incubated at 37°C, 5% CO_2_.

### Growth inhibition assay

Cells were seeded at 3 × 10^3^ cells/well in 96-well plates and treated after 24 h with the indicated compounds. Final DMSO concentration in each well was 0.5% v/v. After 72 h, media was removed and an aliquot of 0.5 mg/mL MTT in media added and incubated for another 2 h, then removed and DMSO added to dissolve the purple formazan crystals. Plates were agitated before absorbance readings were read (570 nm, Tecan Infinite^™^ M200 Pro). The IC_50_ (the concentration of test compound required to inhibit cell growth by 50%) was determined by plotting % viability against logarithmic concentration of test compound (GraphPad Prism 5, San Diego, CA). Experiments were performed in triplicates.

### Detection of apoptosis

Cells were seeded in 6-well plates at 2 × 10^5^ cells/well and treated after 24 h with the indicated compounds. 24 or 48 h later, cells were harvested, washed, and stained with Annexin V-FITC conjugate and propidium iodide according to manufacturer's instructions. Fluorescence was detected on the BD LSRFortessa^®^ Cell Analyzer and analyzed on the FACSDiva Version 6.2 (BD Biosciences, Franklin Lakes, NJ, USA) software. Experiments were done in triplicates.

### Western blotting

Cells were seeded at 5 × 10^5^ cells/plate in 100 mm Petri dishes and treated after 24 h with the indicated compounds. Whole cell lysates were prepared with Cellytic M^®^ buffer or NE-PER^®^ nuclear and cytoplasmic extraction reagents supplemented with phosphatase and protease inhibitors. Protein lysates were subjected to SDS-PAGE and transferred to PVDF membranes. Blocking was performed in 5% non-fat milk followed by probing with primary antibodies. Bands were visualized with Western Bright ECL substrate from Advansta Inc. (Menlo Park, CA, USA) after treatment with secondary HRP-conjugated antibodies.

### Transcription factor assay for NF-κB subunits p59 and p65

Cells were seeded in 6-well plates at 2 × 10^4^ cells/well and treated after 24 hours. 24 or 48 h later, lysates were prepared with NE-PER^®^ nuclear and cytoplasmic extraction reagents. The presence of active p50 or p65 in each fraction was determined with the Pierce NF-κB p50 or p65 transcription factor assay kits according to manufacturer's instructions. Experiments were done in triplicates.

### NF-κB luciferase reporter assay

GloResponse^™^ HEK293 cells were seeded at 3 × 10^4^ cells/well in white-walled 96-well plates for 24 hours before treatment with indicated compounds. After 48 hours, TNFα was added to a final concentration of 20 ng/mL, and incubated for another 5 h. Subsequently, ONE-Glo^™^ Luciferase assay reagent was added, agitated (350 rpm, 3 min) and luminescence read on a plate reader and normalized against the respective viability at each concentration of compound to derive % promoter activity of cells. Experiments were done in triplicates.

### p50 and p65 small-interfering RNA (siRNA) transfection

Cells were seeded in 96-well plates at 3 × 10^3^ cells/well or in 10 mm plates at 5 × 10^5^ cells/plate. After 24 hours, cells were transfected with p50 or p65 siRNA using Lipofectamine 3000^®^ following manufacturer's instructions. ON-TARGETplus^®^ non-targeting siRNAs were used as a negative control.

### Statistical analysis

Data were expressed as mean ± standard deviation. Two-tailed Student's *t*-test was performed (IBM SPSS Statistics v19.0) for pairwise comparison of means and statistical difference. Level of significance was set at *p* < 0.05 unless indicated to be otherwise.

## SUPPLEMENTARY MATERIALS FIGURE AND TABLE


