# Supplementary material for: Dioxonaphthoimidazoliums AB1 and YM155 disrupt phosphorylation of p50 in the NF-κB pathway
Source: Oncotarget. 2016 Feb 10;7(10):11625–36. doi: 10.18632/oncotarget.7299 (PMC4905498; doi:10.18632/oncotarget.7299)
Supplement: Supplementary file 1 [file oncotarget-07-11625-s001.pdf]

Dioxonaphthoimidazoliums AB1 and YM155 disrupt phosphorylation of p50 in the NF-κB pathway

Supplementary Materials

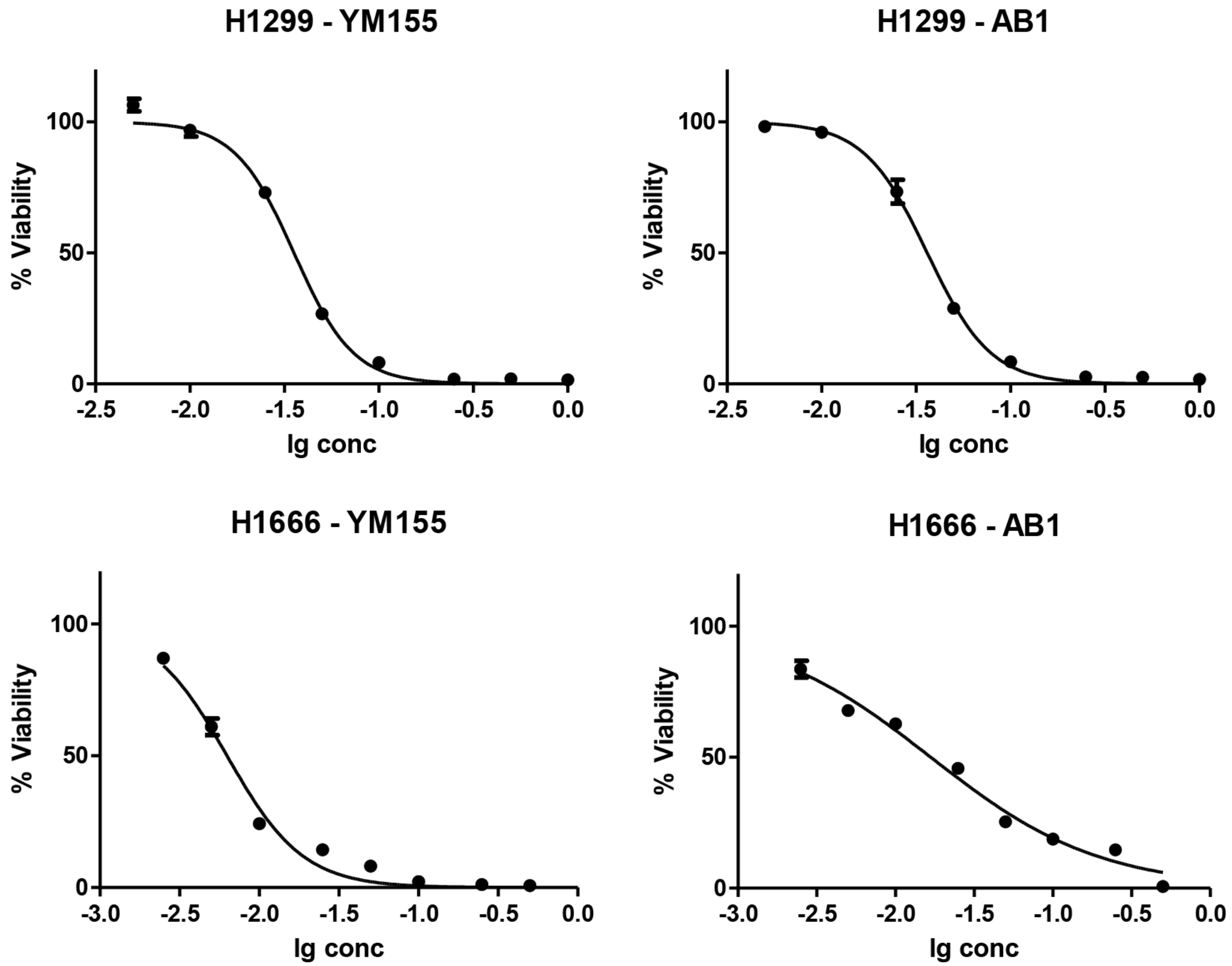

Supplementary Figure S1: Dose-response curves of YM155 and AB1 against H1299 and H1666 using the MTT assay. Each experiment was done in triplicates. Data points represent means with error bars denoting standard deviation.

Supplementary Table S1: Inhibition of respective kinases by AB1 at 10000 nM as a percentage of control determinations were carried out on the scanEDGE^{SM} kinase assay by discoveRx, fremont, CA DiscoveRx, Fremont, CA

Target	AB1	Target	AB1
Gene Symbol	% Ctrl @ 10000 nM	Gene Symbol	% Ctrl @ 10000 nM
AEL1 (E255K)-phosphorylated	64	KIT (D816V)	85
ABL1 (T315l)-phosphorylated	79	KfT (V559D,T670l)	100
ABL1-nonphosphorylated	57	LKB1	86
ABL1-phosphorylated	65	MAP3K4	86
ACVR1B	82	MAPKAPK2	79
ADCK3	99	MARKS	88
AKT1	80	MEK1	81
AKT2	72	MEK2	79
ALK	100	MET	78
AURKA	98	MKNK1	96
AURKB	74	MKNK2	69
AXL	88	MLK1	73
BMPR2	77	p38-alpha	95
BRAF	90	p38-beta	73
BRAF (V600E)	84	PAK1	95
BTK	100	PAK2	93
CDK11	85	PAK4	99
CDK2	87	PCTK1	84
CDK3	85	PDGFRA	100
CDK7	91	PDGFRB	77
CDK9	99	PDPK1	89
CHEK1	82	PIK3C2B	100
CSF1R	100	PIK3CA	100
CSNK1D	81	PIK3CG	93
CSNK1G2	87	PIM1	97
DCAMKL1	56	PIM2	77
DYRK1B	43	PIM3	90
EGFR	79	PKAC-alpha	92
EGFR (L858R}	78	PLK1	73
EPHA2	97	PLK3	92
ERBB2	93	PLK4	93
ERBB4	77	PRKCE	100
ERK1	75	RAFr	100
FAK	87	RET	95
FGFR2	90	RIOK2	57
FGFR3	93	ROCK2	70
FLT3	86	Kin, Dom, 1-N-terminal	60
GSK3B	59	SNARK	100
IGF1R	82	SRC	88
IKK-alpha	75	SRPK3	77
IKK-beta	82	TGFBR1	98
INSR	86	TIE2	72
JAK2 (JH1domain-catalytic)	100	TRKA.	100

JAK3 (JH1 domain-catalytic)	100
JNK1	62
JNK2	56
JNK3	72
KIT	76

TSSK1B	46
JH1 domain-catalytic	100
ULK2	69
VEGFR2	100
YANK3	72
ZAP70	79

97 kinases were screened with members from the serine, threonine and tyrosine kinase families.
